# Effect of Gender on the Knowledge of Medicinal Plants: Systematic Review and Meta-Analysis

**DOI:** 10.1155/2016/6592363

**Published:** 2016-10-04

**Authors:** Wendy Torres-Avilez, Patrícia Muniz de Medeiros, Ulysses Paulino Albuquerque

**Affiliations:** ^1^Laboratory of Ecology and Evolution of Social-Ecological Systems (LEA), Departamento de Biologia, Universidade Federal Rural de Pernambuco, Av. Dom Manoel de Medeiros, s/n, Dois Irmãos, 52171-900 Recife, PE, Brazil; ^2^Ethnobiology and Human Ecology Group, Centro de Ciências Agrárias, Universidade Federal de Alagoas, Rod. BR 104, Km 85, s/n, 57000-100 Rio Largo, AL, Brazil

## Abstract

Knowledge of medicinal plants is not only one of the main components in the structure of knowledge in local medical systems but also one of the most studied resources. This study uses a systematic review and meta-analysis of a compilation of ethnobiological studies with a medicinal plant component and the variable of gender to evaluate whether there is a gender-based pattern in medicinal plant knowledge on different scales (national, continental, and global). In this study, three types of meta-analysis are conducted on different scales. We detect no significant differences on the global level; women and men have the same rich knowledge. On the national and continental levels, significant differences are observed in both directions (significant for men and for women), and a lack of significant differences in the knowledge of the genders is also observed. This finding demonstrates that there is no gender-based pattern for knowledge on different scales.

## 1. Introduction

Science has an interest in identifying patterns of knowledge regarding natural resources on a global scale [1–3]. Albuquerque and Medeiros [4] transpose a macroecological focus to a macro-ethnobiological focus. This transposition suggests that, by using the arguments and concepts of macroecology as a basis for understanding the wealth and abundance of organisms on different scales, both spatial and temporal, in ethnobiology, we can understand how knowledge variables behave on different spatial and temporal scales and thereby advance the understanding of social-ecological systems on both temporal and spatial scales. This understanding supposes that a social-ecological system is the result of the knowledge and use of natural resources in an ecological system of humans who are immersed in a social system [5]. A macro-ethnobiological approach involves the recognition of patterns that are tied to intracultural and intercultural variations in knowledge and the use of natural resources using systematic revision and meta-analysis [4] to advance areas such as nature conservation and bioprospecting [4].

Ethnobiological studies have identified a range of variables that can interfere with the knowledge of natural resources in social-ecological systems. One of the most studied resources is medicinal plant knowledge because it is a structural component of local medical systems [6]; it is the focus of this study. The variables known to affect medicinal plant knowledge include education, occupation, age, gender, and psychosocial variables [7–11].

Gender has been widely studied to understand whether medicinal plant knowledge varies with gender and how this variable influences the structure of local medical systems [9, 10, 12–15]. However, these studies were conducted on a local level. They have not been analysed together to determine whether there is a gender-based pattern in knowledge on a regional or global level that could characterise the influence of gender on the structure of local medical systems on different scales. Such a determination could contribute to the understanding of how predictive variations in knowledge can relate to the gender variable [4]. Albuquerque et al. [16] note the importance of considering variations in knowledge with gender in ethnodirected studies related to the search for medicines. Understanding the variation in knowledge between the genders on different scales is also important for conservation because it enables strategies that consider variations on different scales to be established. Through gender-based differences in resource use, Müller et al. [15] show the importance of including this variable when establishing conservation strategies and public policies.

The results currently indicate that gender-based knowledge is not homogeneous. Many differences have been found in various parts of the world and even within individual countries. Some studies demonstrate that women know more about medicinal plants [12, 14, 17–19]; other studies indicate that men know more [13, 20–22]; and several studies also reveal no difference between the genders in terms of medicinal plant knowledge [7, 10, 23].

In gender-based comparative studies of the knowledge of medicinal plants, the social roles of women are classified as wives and daughters who are in charge of health, diagnosing illnesses, and knowing their prognosis; they are responsible for implementing the first treatments [25, 26]. By contrast, men are in charge of maintaining the household economy and providing resources, leading them to know more about natural resources for other purposes, such as construction [18, 27, 28]. From the perspective of social roles, women should be responsible for medicinal plant knowledge within local medical systems. However, we can observe three directions in the gender-differentiated understanding of medicinal plants.

Given the information above, this study aims to consolidate the results of gender-based studies of medicinal plant knowledge using a systematic review process and a meta-analysis to determine whether there are gender-based patterns in medicinal plant knowledge on different scales (national, continental, and global). We hypothesise that women generally have more medicinal plant knowledge than men on different scales. We hope that this study contributes to an understanding of the influence of the gender variable on local medical systems.

## 2. Materials and Methods

We base our work on the steps for conducting meta-analyses proposed by Cooper [29]: selecting the sources of information; evaluating the information (the inclusion and exclusion criteria and the quality of the studies); and analysing and integrating the results of the studies (a type of meta-analysis). We also follow the recommendations of the PRISMA (Preferred Reporting Items for Systematic Reviews and Meta-Analyses), which is used in biomedical journals but can actually be used for any type of study [24], to improve the clarity and transparency of the systematic review conducted in this study.

It should be noted that in this study we consider gender a variable that involves cultural beliefs and the distribution of resources between the genders on different levels (interactional and individual), which generates patterns of behaviour and organizes practices [30] based on sexual differentiation and sociocultural context [30, 31]. Therefore, gender may influence the variation or pattern of knowledge on different scales (national, continental, and global). Therefore, in this study, we draw from the perspective of gender rather than sex because the concept of biological sex does not include the sociocultural context (see [31]).

It is also important to note that, due to the significant coverage of local knowledge, which includes experiences and knowledge of natural resources accumulated through the relationships of human groups with the environment [32], as well as the lack of gender-based studies that analyse the breadth of local knowledge of medicinal plants, in this systematic review and meta-analysis, the number of species reported for each gender in the selected articles was analysed. We accept the information cited as knowledge (information), the number of known plants in this case, because we cannot determine, based on the data reported in this study, whether this knowledge translates into behaviour or practice.

### 2.1. Selecting the Sources of Information

A search for studies that compare the medicinal plant knowledge of the genders from September 2014 to March 2015 was conducted in databases that include only indexed journals and in specialised journals that publish ethnobiological studies; the studies cited in each of the articles selected for the study were also searched. The keywords used in our first two search strategies included, as a baseline, the word gender due to the objective of our study. In the search for information, only English keywords were used; however, some journals offered information in Spanish or Portuguese. Books and review articles were not considered.

The databases consulted were Scielo (http://www.scielo.org/), Scopus (https://www.scopus.com/), Web of Science (http://apps.webofknowledge.com/), and Science Direct (http://www.sciencedirect.com/); publications from all years included in the databases were considered. In the article search, the following 14 keywords were used: “medicinal plants” AND gender, ethnobiology AND gender, ethnobotany AND gender, ethnomedicine AND gender, “traditional medicinal systems” AND gender, “traditional ecological knowledge” AND gender, “traditional medicine” AND gender, ethnopharmacology AND “medicinal plants”, “medical anthropology” AND gender, “quantitative ethnobotany” AND gender, “quantitative ethnobotany” AND medicinal plants, “intracultural variation” AND “medicinal plants”, “local knowledge” AND “medicinal plants”, and “local knowledge” AND gender.  Table 1 presents an example of the search results for one of the databases consulted as a demonstration of the systematic search for studies in the databases.

Searches of the following specialised journals that publish ethnobiological studies were directed by the keyword “gender”: Economic Botany, the Journal of Ethnobiology and Ethnomedicine, the Latin American Caribbean Bulletin of Medicinal and Aromatic Plants (Boletín Latinoamericano del Caribe de Plantas Medicinales y Aromáticas), Ethnobotany Research and Applications, and the Journal of Ethnopharmacology, Social Science, and Medicine.

### 2.2. Evaluating the Information

#### 2.2.1. Inclusion and Exclusion Criteria

The inclusion and exclusion criteria were based on the characteristics of the published studies and our research objective. They enabled more systematic selection of the studies considered in the analysis.

This investigation included studies that presented the total number of species known by men and women and analyses of the comparisons that the results were based on (mean comparisons, the chi-square statistical test, Student's *t*-test, the Kruskal-Wallis test, and the Mann-Whitney test). The investigation also included studies that analysed the knowledge of men and women in various categories; however, these were only selected when the results of the analysis were reported for each category (only the results for medicinal plants were used), and they were not included when they made a general comparison (in all use categories).

Studies that compared the knowledge of medicinal plants of men and women by relating the numbers of species and diseases treated by each species and studies that only presented comparisons of the diseases treated with plants by each gender were not included because few studies included these analyses, which limited the analysis of the different scales. Studies in which comparisons were performed using diversity indices were not included because the results included other types of information that limited the information compared to most of the selected studies.

Additionally, this study did not include studies in which the gender-based comparison was between specialists (traditional male doctors, midwives, or men and women recognised by their community as holders of knowledge) and nonspecialists (men and women in the community who use the knowledge). Specialists are recognised within communities as wise and knowledgeable about the resources of the region [33]; therefore, comparing the understanding of specialists and nonspecialists generates bias in the results.

Additionally, the investigation included neither studies that compared the genders but only reported whether each interviewee reported using medicinal plant-based remedies nor studies that compared the number of men and women who accepted or refused to use medicinal plant-based remedies.

#### 2.2.2. Quality of the Studies Chosen

To understand the quality of the studies chosen, the studies were classified into three levels of bias risk (low, moderate, and high) based on the quality of the sample selected following Medeiros et al. [34]. Due to the small number of studies with low bias risk, we decided to analyse all of them.

In their meta-analysis of ethnobiological data concerning medicinal plants, Medeiros et al. [35] also found few studies with low bias risk. Systematic reviews and meta-analyses have analysed studies independently of the bias risk of the chosen articles due to the small number of studies with low bias risk [36].

Based on the above information, we decided to use the three different types of meta-analysis described by Cooper [29] because each quantitatively analyses the different ways in which the results were expressed in the selected studies, which allowed us to analyse a greater number of studies. We did not want to compare the results of the three types of meta-analysis; instead, we wanted to observe how the differences between the genders vary when we analysed a larger number of studies.

### 2.3. Analysing and Integrating the Results of the Studies

#### 2.3.1. Data Processing

The following information from each article was registered in a database: the decade of publication, the impact factor of the journal, the country under investigation, whether the study involved one or many usage categories (timber, fuel, food, medicine, etc.), the goal of the study, the population (the total numbers of men and women, specialists, or heads of household), the number of interviewees, the selection criteria for participants (intentional or random), the type of statistical analysis (mean comparison, chi-square statistical test, Student's *t*-test, etc.), and the numbers of species reported by men and women.

Each study was reviewed to obtain the above information. When a study did not present this information, the corresponding field was marked N/A (not available). When the study was conducted in more than one community and presented analyses for each community, the results for each community were considered different results, which resulted in more than one entry to analyse. The studies were classified by country and continent (Africa, the Americas, Asia, Europe, and Oceania).

#### 2.3.2. Data Analysis

The data were analysed on three scales (national, continental, and global) based on the premises of macroecology, in which the influence of certain variables on species richness depends on the scale [37]. The data were analysed at the different scales using the three types of meta-analysis. On the national level, only two countries were analysed due to the paucity of studies for each country; only Ethiopia and Brazil presented more than three studies.

*(1) Simple Count Meta-Analysis*. This meta-analysis consisted of counting the results of the studies without considering whether they were statistically significant, that is, only considering the results without any statistical analysis [29]. For example, if one study showed that women identified significantly more medicinal plants than men but that men identified, on average, 40 species and women identified 100, then, independently of their significance, the total numbers of species identified by each gender were considered; thus, the numbers of species identified by men and women according to each study were obtained. For this meta-analysis, we included studies that reported the numbers of medicinal plants identified by men and women. The statistical analyses used for each scale varied. On the global level, a chi-square analysis was used. On the national and continental levels, a contingency table analysis using Fisher's exact test was used because some results had values that were smaller than five. The statistical analyses were conducted using R version 2.13.2 [38].

*(2) Vote Count Meta-Analysis*. The vote count meta-analysis was based on counting the statistical results, regardless of whether they were significant in support of the hypothesis being tested, and the results that were not significant [29]. For example, in the simple count meta-analysis, it did not matter whether men identified an average of 40 species and women identified 100 species; what mattered here was the significance. From each study, it was determined whether men identified significantly more medicinal plants than women, whether women identified significantly more medicinal plants than men, or whether there were no significant differences in the knowledge of the genders. Therefore, for this meta-analysis, the statistical results of the comparison between the numbers of species identified by the men and women of the communities under study in each of the selected articles were analysed. The analyses performed on the different scales were the same as those performed in the simple count meta-analysis.

*(3) Effect Size Meta-Analysis*. The effect size meta-analysis consisted of combining the statistical results of each study and standardising them using the “effect size.” The effect size is the degree to which a phenomenon manifests in the population, which is related to statistical values. The calculation of the effect size standardises the statistical results using the “test *d*” (standardised mean difference) or the “test *r*” (correlation coefficient), which transformed the data into comparable values that are independent of the original statistical test [29].

The studies evaluated the differences in knowledge between the genders using the chi-square statistical test, Student's *t*-test, the Mann-Whitney test, and mean comparisons. To calculate the effect size of the statistical results, the values of the mean, the standard deviation, the *t*-test, the chi-square, and the number of participants analysed in each study were used. The information used depended on the statistics used in the study. The calculations were performed using the George Mason University website [39].

Once the effect sizes were obtained for the results of each study, we used a random effect model to determine whether global differences existed based on the values for the studies that favoured knowledge for men and/or women. Subsequently, the effect sizes were analysed using a mixed effect model to analyse the data by continent. This meta-analysis was not performed by country due to the limited number of studies in each country (Ethiopia and Brazil) for which effect sizes could be calculated. The statistical analyses were conducted using R version 2.13.2 (The R Foundation for Statistical Computing, 2011) with the Rcmdr, NCStats, metafor, and vegan packages and *α* = 0.05.

## 3. Results 

**Figure 1:**
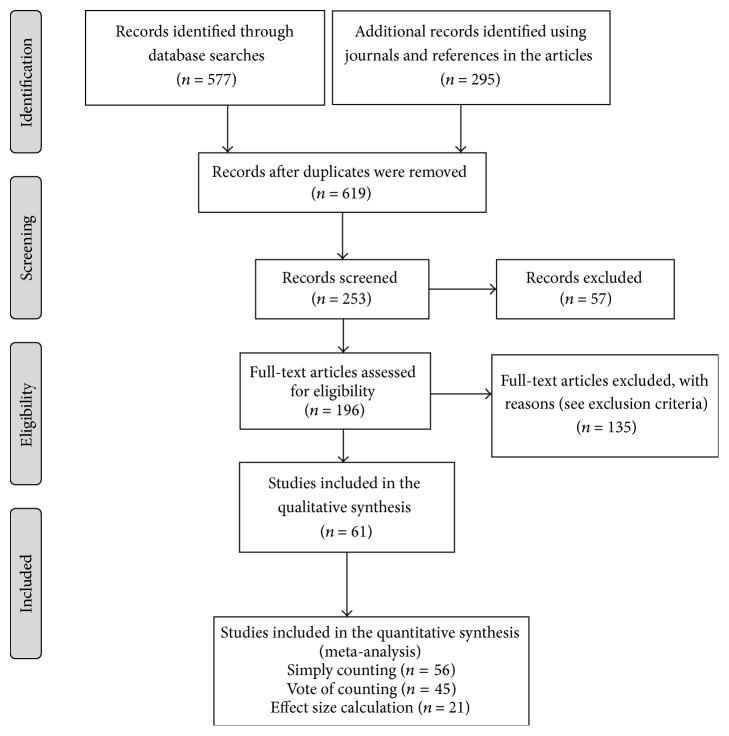
Flowchart summarising the selection of ethnobiological studies of gender and medicinal plant knowledge. Format proposed by Moher et al. [24].

Of the 196 articles reviewed, only 61 are included in the analysis after the exclusion criteria were applied; these are the only studies involving gender-based comparisons of knowledge conducted to date that can be combined to analyse the variable of gender in a general manner (Appendix). These articles pertain to 26 countries from four continents (Africa, the Americas, Asia, and Europe). Brazil and Ethiopia provide the most articles at 13 and nine, respectively. From the 61 articles selected, 65 entries are obtained because some of the articles present results for more than one community (Table 2). Therefore, the numbers of articles and entries analysed in each meta-analysis vary. In the simple count meta-analysis, 56 articles are selected, and 60 entries are analysed; in the vote count, 45 articles and 47 entries are analysed; and in the effect size calculation, 21 articles and 21 entries are analysed (Figure 1).

Upon analysing the 61 articles selected based on the quality of the selection of the analysed samples, following the proposal of Medeiros et al. [34], 85% present a high bias risk, 7% a moderate bias risk, and 8% a low bias risk. The sample selection in the articles is primarily based on the total community population and the number of residences (Table 3).

With regard to the analysis on the global level, the results reveal no significant differences in the knowledge of men and women in any of the three types of meta-analysis (Table 4).

The results obtained on the continental level indicate significant differences in the knowledge of men and women in the results of the simple count and vote count meta-analyses. In both meta-analyses, the African and American continents present the most studies. For the African continent, more studies demonstrate that men know more in both meta-analyses. For the American continents, more studies in the simple count indicate that women know more, and most studies in the vote count reveal no significant difference in the knowledge of the two genders. The results of the effect size meta-analysis indicate no significant difference between the genders (Table 5).

The simple count and vote count meta-analyses by country demonstrate significant differences in the knowledge of men and women; women know more in Brazil, whereas men know more in Ethiopia (Table 6).

## 4. Discussion

Because significant differences are detected only on the national and continental levels and not on the global level, our results do not support the proposed hypothesis. Our results suggest that these differences are only observable on smaller scales and that the differences are not unidirectional. Either men or women can have more knowledge, or there can be no difference in their knowledge. In their descriptive study analysing the local knowledge of natural resources of the genders, Pfeiffer and Butz [40] suggest that the difference between the genders can be in these three directions; by contrast, this study, which considers three types of meta-analysis and different scales, suggests that the difference in local knowledge between the genders can vary with the scale (national, continental, or global).

The results of this study demonstrate that the supposition that women know more because they are homemakers and are responsible for the health of the family cannot always be applied on different scales, which may reflect the heterogeneity of the strategies for the division of labour that do or do not favour a specific gender available to communities; these strategies are more homogenous on the local scale.

This heterogeneity of strategies is also observed in the knowledge of other resources, such as the knowledge of plants for firewood. In Brazil, men have been shown to know more than women because men are responsible for collecting plants; however, women can be familiar with them for their cooking uses [41]. Additionally, women in Africa frequently collect plants for firewood as one of the domestic activities they are responsible for [42, 43]. In some places, the diameter of the plants collected for firewood varies by gender, which may be related to the tool used to obtain the resource [42].

Because the differences found between the genders can be a product of the heterogeneity of strategies of division of labour in the communities, it is important to note that the division of labour is related to variables such as age, race, caste, class, and ethnicity [44]. This also holds for social restructuring as a result of globalisation and responding to political, economic, cultural, and technological changes; therefore, it influences the construction of more dynamic and less directed roles for a specific gender [26]. However, recent ethnobiological studies of gender do not include variables such as the division of labour in their analyses; therefore, this line of reasoning cannot be used as a universal argument.

Doyal [26] emphasises that because the division of labour is the product of many variables, it cannot be considered universal when the particular characteristics of each community are considered. The results of this study and the argument that our hypothesis sustains demonstrate that, on a more local (national) level, homogeneity in the division of labour may be in accordance with the significant differences in knowledge between the genders in Brazil and Ethiopia.

In the macroecology in which macro-ethnobiology is based, it has been argued that the richness of species varies in relation to the scale as a product of the variation of certain factors that are present on each scale [37]. However, in the case of macroscale variations in local knowledge by gender, one cannot argue based on intervening factors. Most studies do not analyse such factors and only quantify knowledge, which suggests that its variation depends on the division of labour, which is not analysed [45].

Conversely, some studies, such as the ones conducted in Ethiopia that were analysed in this investigation, lack evidence-based argumentation. These studies argue that differences in knowledge between men and women reflect the social norms of the communities under study, in which men are supposed to obtain medicinal knowledge [13, 20, 21]. These types of arguments generate doubts and other questions because there may be other directions from which one may learn within the learning dynamic. For example, women, followed by men, may transmit knowledge more frequently, as reported in Brazil [46]. This approach considers the existence of gender-based learning models in which gender roles are well defined and that propose that children tend to learn with others of the same sex. However, this depends on the information in question. For example, when children want to know something related to health, they tend to ask women, who are more closely related to this role than men [47, 48].

In macro-ethnobiological studies of medicinal plants and gender, although information that describes the community under study can contribute to the explanation of the difference between the genders, most studies do not include information such as the type of community (nonindigenous or indigenous), the area of the community (rural or urban), the level of dependence on natural resources, the type of subsistence, and social norms.

Based on the results obtained in this study, future studies of gender that analyse variations in knowledge between the genders should be more grounded in the variables that directly influence the dynamics of knowledge of each gender. It has been suggested that the variations in knowledge between the genders can be influenced by specific factors, such as the transmission of knowledge between the genders, gender-based differences in social networks, cultural roles and spiritual taboos that influence social beliefs, and the norms for each sex, which involve different components of managing and not managing the ecosystem, differences in access to resources, and sex-based differences in access to formal and external knowledge [40]. These factors can be influenced by differences in the behaviour of the sexes. This approach can use the biosocial model proposed by Wood and Eagly [49], which supposes that the differences in behaviour between men and women depend on factors such as the physical specialisation of each gender, the economic attributes of the society, the social structure, and ecological considerations.

### 4.1. Suggestions on What to Report and Evaluate in Future Gender-Based Studies of Medicinal Plant Knowledge

With the aim of facilitating studies using systematic reviews and meta-analyses and considering the limitations that we faced while conducting this study, we recommend including the following in published studies of gender:

(i) In the statistical results, present the *p* value, the standard deviation, and the results of each test.
(ii) Conduct representative selection of the total sample using the total, “*N*”, and the sample size, “*n*”.
(iii) Report the richness of species known by each gender independently of whether these data are necessary for statistical analyses that are directly related to the objectives.
(iv) Include more specific information in the description of the community under study, such as whether it is indigenous, whether it is established in an urban or rural area, the level of dependence on natural resources, the type of subsistence, and information on certain social norms.
(v) In the discussion of results, note a possible reason for the difference in knowledge between the genders or lack thereof, even when this comparison is not part of the objectives.

We recommend that future studies comparing the knowledge of the genders evaluate the dynamics of possible factors that can explain the variation in knowledge so that these factors can be considered in future studies on the national, continental, and global levels. This information would then help distinguish the reasons for the differences on different scales, in addition to viable results, to establish conservation strategies and directed bioprospecting studies that consider the importance of every social actor to the health-related use of plants.

### 4.2. Limitations of the Study

This systematic review represents an effort to synthesise the results of studies regarding the medicinal plant knowledge of men and women. However, this study has the following limitations:

(i) Studies conducted in different countries, which would strengthen the results on the different scales, are lacking.
(ii) The diversity of the research groups that study the relationship between gender and medicinal plant knowledge is low; the studies analysed originate from one research group, which can bias the results.
(iii) The information on the analyses offered in the publications and in the methodological specifications, such as the criteria for selecting the *N* under study, is limited.
(iv) Only 34% of the studies that compared knowledge between the genders provided the information necessary to perform a meta-analysis based on the effect size, which is more robust.

## Figures and Tables

**Table 1:** Example of the results of searches for ethnobiological studies on gender and medicinal plant knowledge in the Science Direct database.

Keywords	Search results	Records selected	Records included	Records duplicated in other search results
“medicinal plants” AND gender	1,183	35	19	16
ethnobiology AND gender	222	16	4	12
ethnobotany AND gender	338	31	5	26
ethnomedicine AND gender	268	11	0	11
“traditional medicinal systems” AND gender	7	1	0	1
“traditional ecological knowledge” AND gender	195	8	2	6
“traditional medicine” AND gender	1,640	23	1	22
ethnopharmacology AND “medicinal plants”	8,524	14	0	14
“medical anthropology” AND gender	1,261	2	0	2
“quantitative ethnobotany” AND gender	56	10	0	10
“quantitative ethnobotany” AND medicinal plants	183	11	0	11
“intracultural variation” AND “medicinal plants”	1	1	0	1
“local knowledge” AND “medicinal plants”	405	20	4	16
“local knowledge” AND gender	1,859	8	0	8

**Table 2:** Number and list of studies by continent and country.

Continent	Country	Number of studies	Studies
Africa		**24**	
	Burkina Faso	2	[50, 51]
	Ethiopia	9	[13, 20, 52–55]; [21] (three communities)
	Kenya	3	[56–58]
	Lesotho	1	[59]
	Madagascar	2	[60, 61]
	Mozambique	1	[62]
	Niger	3	[8, 15, 63]
	South Africa	1	[64]
	Tanzania	2	[65, 66]

America		**25**	
	Brazil	16	[9, 10, 12, 17, 22, 67–77]
	Dominica	1	[78]
	Mexico	5	[18, 79]; [80] (three communities)
	Peru	2	[23, 81]
	Venezuela	1	[82]

Asia		**10**	
	India	2	[83, 84]
	Indonesia	1	[27]
	Manus Island	1	[85]
	Pakistan	2	[86, 87]
	Palestine	1	[88]
	Philippines	1	[19]
	Thailand	2	[89, 90]

Europe		**6**	
	Austria	1	[14]
	Czech Republic	1	[91]
	Italy	1	[92]
	Serbia	2	[93, 94]
	Spain	1	[28]

**Table 3:** Percentage of studies in each risk category based on the quality of the sample [34]. *U* is the total population size, and *N* is the sample size in relation to *U*.

Origin of the sample	Sample	Risk level	Percentage of studies
(1) When the sample is determined by the total number of people or an age interval	(b) When *N* is less than 80% of the necessary value for its representation with a margin of error of up to 5%.	High	10
	(b) When *N* is less than 80% of the necessary value for its representation with a margin of error of up to 5%. (c) When there is no information about *U* or *N*.	High	2
	(c) When there is no information about *U* or *N*.	High	56
	(a) When *U* is equal to *N*.	Low	3
	(b) When *N* is representative of *U* with a randomized sample and a margin of error of up to 5%.	Low	3
	(a) When *N* is extracted from *U* with a randomized sample and a margin of error greater than 5% but less than 10%.	Moderate	3

(2) When the sample is based on heads of household (one or two per household)	(b) When *N* is less than 80% of the value necessary to represent the heads of household with a margin of error of up to 5%.	High	2

(3) When the sample is based on households	(b) When *N* is less than 80% of the value necessary to represent the households with a margin of error of up to 5%.	High	3
	(b) When *N* is less than 80% of the value necessary to represent the households with a margin of error of up to 5%. (c) When there is no information on the number of households or *N*.	High	2
	(c) When there is no information on the number of households or *N*.	High	7
	(b) When, in the representative number of homes, one of the household members is interviewed, with a randomized sample and a margin of error of up to 5%.	Low	2

(4) When the sample is intentionally focused on an interest group (e.g., midwives, herbalists, or local specialists)	(d) In cases of local specialists, when there is no indication of the total, but the snowball technique is used to select the principal people with knowledge.	Moderate	2

(5) When participatory methods are used	(b) When there is no information about the size of the population or group in question, but information about the number of participants is provided.	Moderate	2

(6) Diffuse selection criteria	(a) When there is no information on *N* or *U*.	High	5

A total of 80% of the complete (100%) sample is used with a margin of error of less than 5%.

**Table 4:** Analysis of the medicinal plant knowledge of the two genders on the global level.

Type of meta-analysis	Total	Number per gender	Results
Simple count	T = 60	W = 33 M = 27	*χ* ^2^ = 0.6 *p* = 0.4386

Vote count	T = 47	WM = 12 MM = 12 ND = 22	*χ* ^2^ = 3.87 *p* = 0.14

Effect size calculation	T = 21	W = 14 M = 7	SD *p* ≥ 0.05

T: total studies analysed.

W: number of studies in which women know more.

M: number of studies in which men know more.

WM: number of statistically tested studies in which women know more.

MM: number of statistically tested studies in which men know more.

ND: number of statistically tested studies in which there is no difference in knowledge between the genders.

**Table 5:** Analysis of the medicinal plant knowledge of the two genders on the continental level.

Type of meta-analysis	Total studies	Africa	America	Asia	Europe	Results
Simple count	T = 60	W = 5 M = 18	W = 18 M = 3	W = 5 W = 5	W = 5 M = 1	*p* ≤ 0.0001

Vote count	T = 47	WM = 1 MM = 12 ND = 7	WM = 6 MM = 1 ND = 10	WM = 3 MM = 0 ND = 3	WM = 2 MM = 0 ND = 2	*p* ≤ 0.0001

Effect size calculation	T = 21	W = 5 M = 4	W = 8 M = 2	M = 1	W = 1	SD *p* ≥ 0.05

T: total studies analysed.

W: number of studies in which women know more.

M: number of studies in which men know more.

WM: number of statistically tested studies in which women know more.

MM: number of statistically tested studies in which men know more.

ND: number of statistically tested studies in which there is no difference in knowledge between the genders.

**Table 6:** Analysis of the medicinal plant knowledge of the two genders on the national level.

Type of meta-analysis	Total	Brazil	Ethiopia	Results
Simple count	T = 22	W = 12 M = 1	W = 0 M = 9	*p* ≤ 0.0001

Vote count	T = 21	WM = 4 MM = 1 ND = 7	WM = 0 MM = 8 ND = 1	*p* ≤ 0.0001

Effect size calculation	—	—	—	—

T: total studies analysed.

W: number of studies in which women know more.

M: number of studies in which men know more.

WM: number of statistically tested studies in which women know more.

MM: number of statistically tested studies in which men know more.

ND: number of statistically tested studies in which there is no difference in knowledge between the genders.

**Table 7:** List of studies analysed.

Study	Country of study	Continent	Simple count	Vote count	Effect size	Bias criteria
Ayantunde et al. [8]	Niger	Africa	MM	ND	0.1184	1Ab
Beltrán-Rodríguez et al. [79]	Mexico	America	WM	ND	0.3158	1Ab
Da Silva and Proença [67]	Brazil	America		ND		1Ab
Kidane et al. [21]^*∗*^	Ethiopia	Africa	MM	SM		1Ab
Voeks and Leony [17]	Brazil	America	WM	SW		1Ab
Zucchi et al. [68]	Brazil	America	WM			1Ab
Begossi et al. [69]	Brazil	America	WM		0.3165	1Ab-1Ac
Augustino et al. [65]	Tanzania	Africa	MM	SM		1Ac
Bruschi et al. [62]	Mozambique	Africa	WM	SW	0.772	1Ac
Estrada-Castillón et al. [80]^*∗*^	Mexico	America	WM	ND		1Ac
Giday et al. [20]	Ethiopia	Africa	MM	SM		1Ac
Giday et al. [52]	Ethiopia	Africa	MM	SM		1Ac
Giday et al. [53]	Ethiopia	Africa	MM	SM		1Ac
Khuankaew et al. [89]	Thailand	Asia	WM	ND		1Ac
Kristensen and Balslev [50]	Burkina Faso	Africa	MM	ND		1Ac
Bisht et al. [83]	India	Asia	MM	ND	−0.0483	1Ac
Lulekal et al. [54]	Ethiopia	Africa	MM	ND	0.1822	1Ac
Miranda et al. [70]	Brazil	America		ND		1Ac
Müller et al. [15]	Niger	Africa	WM	ND	1.0977	1Ac
Nanyingi et al. [56]	Kenya	Africa		ND		1Ac
Ngari et al. [57]	Kenya	Africa	MM	SM	−0.6512	1Ac
Ong and Kim [19]	Philippines	Asia	WM	SF		1Ac
M. B. Quinlan and R. J. Quinlan [78]	Dominica	America	WM			1Ac
Qureshi et al. [86]	Pakistan	Asia	MM			1Ac
Reyes-García et al. [28]	Spain	Europe	WM	SF		1Ac
Santos et al. [71]	Brazil	America	WM	SF		1Ac
Šavikin et al. [93]	Serbia	Europe	WM	ND	0.2556	1Ac
Savo et al. [92]	Italy	Europe	WM			1Ac
Schunko et al. [14]	Austria	Europe	WM	SF		1Ac
Semwal et al. [84]	India	Asia	MM			1Ac
Silva et al. [72]	Brazil	America	WM		0.4867	1Ac
Silva et al. [9]	Brazil	America	WM	ND	−0.205	1Ac
Sop et al. [51]	Burkina Faso	Africa	WM	ND	0.0976	1Ac
Souto and Ticktin [82]	Venezuela	America	—	ND		1Ac
Srithi et al. [90]	Thailand	Asia	WM	SF		1Ac
Stagegaard et al. [23]	Peru	America	MM	—		1Ac
Teklehaymanot [55]	Ethiopia	Africa	MM	SM	−7.0521	1Ac
Voeks [12]	Brazil	America	WM	SF		1Ac
Warui [58]	Kenya	Africa	MM	SM		1Ac
Zank and Hanazaki [73]	Brazil	America	WM	ND	0.2751	1Ac
Zlatković et al. [94]	Serbia	Europe	MM	ND		1Ac
Alencar et al. [74]	Brazil	America	WM	ND	0.0126	1Ba
Caniago and Siebert [27]	Indonesia	Asia	WM			1Ba
Lyon and Hardesty [60]	Madagascar	Africa	WM			1Bb
Sawalha et al. [88]	Palestine	Asia	WM	SW		1Bb
Albuquerque et al. [22]	Brazil	America	MM	SM	−0.22062	1Ma
Teklehaymanot and Giday [13]	Ethiopia	Africa	MM	SM	−1.9016	1Ma
Luoga et al. [66]	Tanzania	Africa	MM	—	−3.1018	2Ab
Camou-Guerrero et al. [18]	Mexico	America	WM	SW	0.6552	3Ab
Luziatelli et al. [81]	Peru	America	WM	SW		3Ab
de Almeida et al. [75]	Brazil	America	WM	SW	0.443	3Ab-3Ac
Letšela et al. [59]	Lesotho	Africa	MM	SM		3Ac
Andriamparany et al. [61]	Madagascar	Africa	WM			3Ac
Guimbo et al. [63]	Niger	Africa	MM	ND		3Ac
Merétika et al. [76]	Brazil	America	WM	ND	0.5362	3Ac
de Almeida et al. [10]	Brazil	America	—	ND		3Bb
de Brito and de Senna-Valle [77]	Brazil	America	WM			4Md
Sher et al. [87]	Pakistan	Asia	MM			6Mb
Case et al. [85]	Papua New Guinea	Asia	MM	ND		7a
Dovie et al. [64]	South Africa	Africa	MM			7a
Knotek et al. [91]	Czech Republic	Europe	WM			7a

^*∗*^Study of three communities.

MM: men know more; WM: women know more.

SW: significant for women; SM: significant for men; ND: no significant difference.

In the effect size column, a positive value indicates that women know more and a negative value indicates that men know more.

Bias criteria

(1) When the sample is extracted from the total number of people or from an age group:

A = high; b = when *N* is less than 80% of the value necessary for its representation with a margin of error of up to 5%.

M = moderate; a = when  *N* is extracted from *U* with randomisation and a margin of error that is greater than 5% but less than 10%.

B = low; a = when *U* is equal to *N*; b = when *N* is representative of *U* with a randomised sample and a margin of error of up to 5%.

(2) When the sample is based on heads of household (one or two per home):

A = high; b = when *N* is less than 80% of the value necessary to represent the heads of household with a margin of error of up to 5%.

(3) When the sample is based on households:

A = high; c = when there is no information about the number of households or *N*; b = when *N* is less than 80% of the value necessary to represent the households with a margin of error of up to 5%.

B = low; b = when in the representative number of households one of the household members is interviewed, with a randomised sample and a margin of error of up to 5%.

(4) When the sample is intentionally focused on an interest group (e.g., midwives, herbalists, or local specialists):

M = moderate; d = in cases of local specialists, when there is no indication of the total, but the snowball technique is used to select the principal people with knowledge.

(5) When participatory methods are used:

M = moderate; b = when there is no information about the size of the population or the group in question, but information about the number of participants is provided.

(6) Diffuse selection criteria:

A = high; a = when there is no information on *N* or *U*.

## Appendix

See Table 7.
